# An epidemiological survey of HBV infection and low-level HBsAg in military camps in eastern China: Erratum

**DOI:** 10.1097/MD.0000000000014273

**Published:** 2019-01-25

In the article, “An epidemiological survey of HBV infection and low-level HBsAg in military camps in eastern China”,^[1]^ which appeared in Volume 97, Issue 38 of *Medicine*, the funding number for the support from Natural Science Foundation of Zhejiang Province appeared incorrectly and should be No. LY15H200001.
